# Tumor mutation load better predicts the prognosis of patients treated with immune checkpoint inhibitors in upper gastrointestinal cancers: A systematic review and meta‐analysis

**DOI:** 10.1002/cnr2.1959

**Published:** 2024-01-11

**Authors:** Chenghao Ma, Qiong Teng, Liang Shang, Fengying Du, Leping Li

**Affiliations:** ^1^ Department of Gastrointestinal Surgery, Shandong Provincial Hospital, Cheeloo College of Medicine Shandong University Jinan China; ^2^ Department of Gastrointestinal Surgery Shandong Provincial Hospital Affiliated to Shandong First Medical University Jinan China

**Keywords:** immune checkpoint inhibitor, meta‐analysis, prognosis, tumor mutation load, upper gastrointestinal cancers

## Abstract

**Background:**

Tumor mutational load (TML) has emerged as a potential biomarker for multiple solid tumors. However, data on its prognostic impact on upper gastrointestinal (UGI) cancer are limited. Therefore, the aim of this systematic review and meta‐analysis was to assess the prognostic value of TML for the survival of patients with UGI cancer.

**Method:**

A comprehensive search of the PubMed, Embase, Cochrane Library, and Web of Science databases was conducted up to February 13, 2023. Eleven studies met our inclusion criteria. Hazard ratios (HRs) for progression‐free survival and overall survival and their 95% confidence intervals (CIs) were calculated. Subsequently, the combined HR and its 95% CI were calculated for UGI tract cancers in the high and low TML groups. *I* ^2^ statistics and *p*‐values were used to evaluate heterogeneity. Publication bias, sensitivity, and subgroup analyses were performed to determine sources of heterogeneity.

**Results:**

In total, 932 patients with UGI tract cancer from 11 publications were included. The high TML group treated with immunotherapy showed significantly improved overall survival (HR = 0.68; 95% CI: 0.53, 0.86; *p* = .001) and progression‐free survival (HR = 0.74; 95% CI: 0.58, 0.95; *p* = .020) compared with the low TML group.

**Conclusion:**

Our study demonstrated that patients with UGI tumors and higher TML have a better prognosis with immunotherapy, suggesting that TML is a promising predictive biomarker for immunotherapy.

**Registration:**

The study protocol was registered with the International Prospective Register of Systematic Reviews (PROSPERO Registration No: CRD42023405596).

## 1 INTRODUCTION

Upper gastrointestinal (UGI) malignancies include gastric, gastroesophageal junction, and esophageal tumors. According to the World Health Organization's global cancer statistics, 1.09 million people are diagnosed with stomach cancer and 600 000 with esophageal cancer every year, making it the fourth most common cancer worldwide. The incidence of gastric cancer (GC) deaths was 770 000 and that of esophageal cancer deaths was 540 000, ranking second in total mortality among all tumors.^1^ Despite improvements in survival rates, the 5‐year survival rate for advanced UGI tumors remains unsatisfactory, with cytotoxic chemotherapy being the primary treatment option.^2^, ^3^ Immunotherapies have proven to be a promising option, changing the standard of care for some solid tumors.^4^, ^5^ Due to the low overall response rate to immunotherapy in UGI cancers, there is a need for selection of patients who may be eligible for immunotherapy.^6^, ^7^ Several biomarkers have been reported to predict immunotherapy response, including programmed death ligand 1 (PD‐L1), microsatellite instability (MSI), tumor mutational load (TML), and tumor‐infiltrating lymphocytes, among others.^8^, ^9^, ^10^ TML refers to the total number of mutations per megabase of tumor tissue which can be detected by either next‐generation sequencing (NGS) or whole‐exome sequencing (WES), and reflects the overall burden of tumor antigens. The fundamental principle of immunotherapy is to stimulate and enhance host T lymphocytes to eliminate tumors. Theoretically, a higher TML in cancer cells makes them more likely to be recognized by the immune system, thereby improving the effectiveness of immunotherapy.^11^, ^12^, ^13^ According to previous studies, non‐small cell lung cancer and melanoma have higher tumor mutation loads than other tumors, whereas UGI tract tumors do not have very high tumor mutation loads.^14^, ^15^ Although there has been considerable progress in the study of TML as a predictive biomarker in non‐small cell lung cancer and melanoma, its application to UGI cancer remains incomplete.^16^, ^17^ Therefore, the aim of this systematic review and meta‐analysis was to evaluate the prognostic value of TML in UGI cancers treated with immune checkpoint inhibitors (ICIs) on the basis of the latest data.

## 2 MATERIALS AND METHODS

This systematic review and meta‐analysis were conducted in accordance with the Preferred Reporting Items for Systematic Reviews and Meta‐Analysis guidelines provided in Supplementary File S1.^18^ This systematic review and analysis were registered in the International Prospective Register of Systematic Reviews (CRD42023405596, https://www.crd.york.ac.uk/PROSPERO).

### 2.1 Search strategy

A comprehensive search of PubMed, Embase, Cochrane Library, and Web of Science databases was performed for the period of time up until February 13, 2023, using the following search terms: (esophageal neoplasms OR gastroesophageal neoplasms OR stomach neoplasms) AND (mutation burden OR mutation load OR tumor mutational burden OR TML) AND (immunotherapy OR immune checkpoint inhibitor OR ICI OR immune checkpoint blocker OR ICB OR PD‐1 OR PD‐L1 OR CTLA‐4). The detailed search strategies are provided in Supplementary File S2.

### 2.2 Study selection

Two investigators (CHM and QT) independently searched and screened the studies and resolved disagreements through discussion and consensus. Subsequently, the titles and abstracts of the studies were assessed for eligibility according to the inclusion criteria, after removing duplicate reports.

1. Patients were diagnosed with UGI cancer based on pathological results.
2. A well‐defined TML cutoff was used to stratify the subjects into two groups: those with high TML and those with low TML.
3. Studies should evaluate the predictive value of TML for the prognosis of various ICIs, including anti‐PD‐1, anti‐PD‐L1, anti‐CTLA‐4, and their combination therapies.
4. For TML‐related overall survival (OS) or progression‐free survival (PFS), studies should report the hazard ratio (HR) and its 95% confidence interval (CI). Studies should provide Kaplan–Meier curves or raw OS or PFS data to allow the calculation of relevant metrics if these conditions cannot be met.
5. Case reports, reviews, conference abstracts, editorials, letters, animal studies, and studies with incomplete data were excluded from the analysis. Only studies written in English and which met the eligibility criteria were included after a full‐text review.

### 2.3 Data extraction

Data extracted from eligible studies included author, publication year, region, tumor type, patient number, ICI type, sample source, TML sequencing method, TML cutoff value, median TML and range, survival outcome, HRs, and 95% CIs.

### 2.4 Quality assessment

The studies included in this review were cohort studies and randomized controlled trials. The quality of the cohort studies was assessed using the Newcastle‐Ottawa Scale (NOS), which is divided into three dimensions with a score range of 0–9. Studies were categorized into three levels based on their cumulative scores: high quality,^8^, ^9^ moderate quality,^5^, ^6^, ^7^ and low quality (0–4).^19^ In particular, the quality of only the randomized controlled trials that included patients treated with pembrolizumab in this analysis was assessed using the NOS.^20^

### 2.5 Statistical analysis

By comparing the PFS and OS between the high and low TML groups, we verified the predictive value of TML in the prognosis of immunotherapy in UGI cancers using HR. Cox proportional hazards regression models were used to calculate HRs and corresponding 95% CIs for PFS and OS using IBM SPSS Statistics 25 for studies that provided raw data. We used the Engauge Digitizer to extract survival data and the Tierney et al. program file to calculate HRs and 95% CIs for studies that presented Kaplan–Meier curves without HRs for PFS and OS. RevMan 5.4.1 was used to estimate the summary HR, 95% CI, and *p*‐values.^21^

STATA 13 was used to assess the heterogeneity using the *I* ^2^ statistic and *p*‐values. Fixed‐ and random‐effects models were applied to the meta‐analyses with low and high heterogeneity, respectively. Heterogeneity levels were classified as low (25% < *I* ^2^ < 50%), moderate (50% < *I* ^2^ < 75%), or significant (*I* ^2^ > 75%).^22^ If there was significant heterogeneity, the source was further investigated by assessing publication bias, conducting sensitivity analyses, and performing subgroup analyses. To ensure that a sufficient number of eligible studies were included, we conducted a funnel plot and an Egger's test to assess publication bias, with *p* > .05 indicating the absence of publication bias.^23^ To assess the effect of excluding individual studies on summary HR, sensitivity analyses were performed. The region, tumor type, ICI type, sample source, TML sequencing method, and TML cutoff value were set as subgroups for subgroup analysis.

## 3 RESULTS

### 3.1 Literature search

**FIGURE 1:**
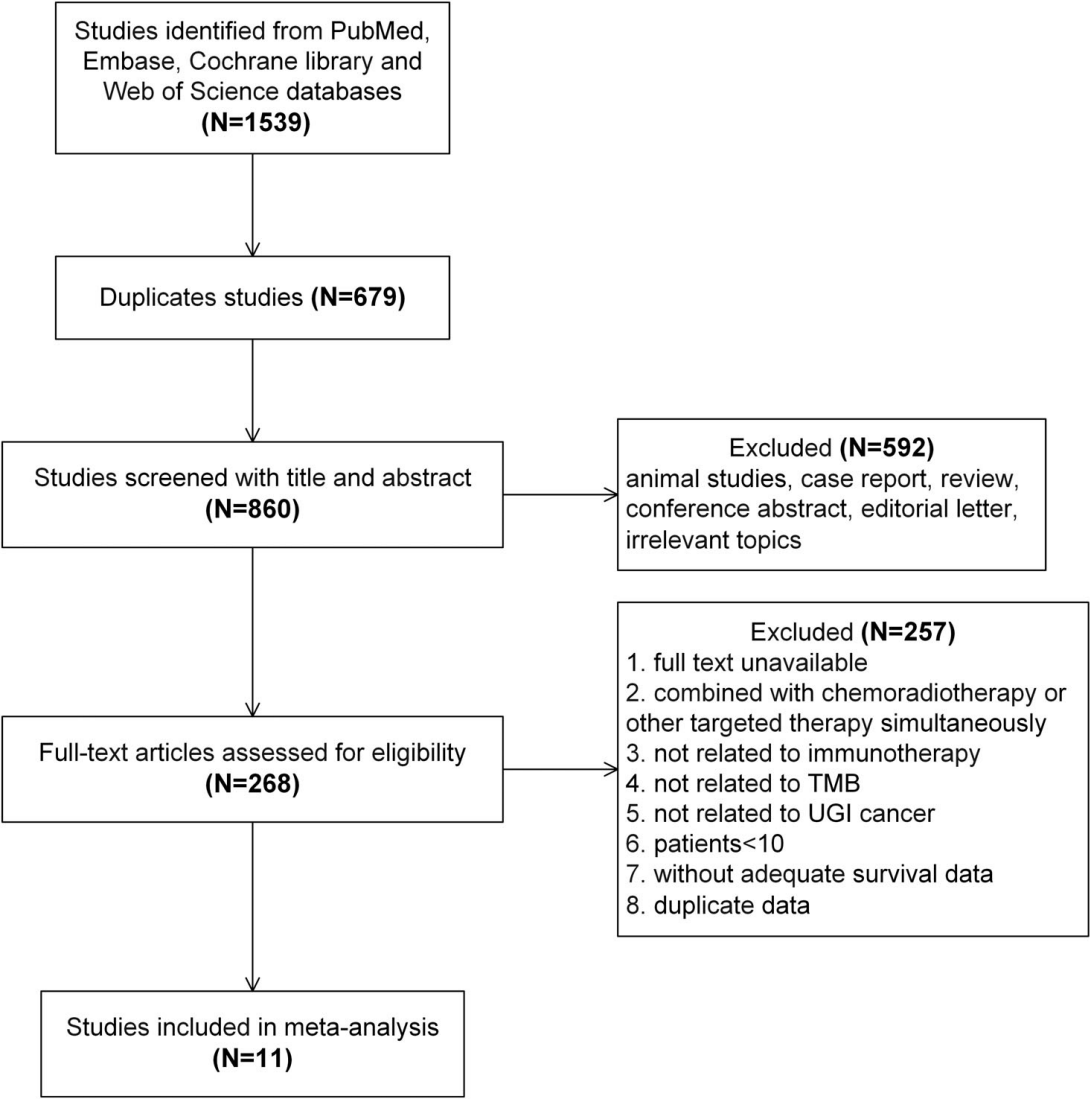
The flow diagram of the study selection process. TMB, tumor mutational burden; UGI, upper gastrointestinal.

After removing 679 duplicate studies, a total of 860 studies were retrieved from the Web of Science, Embase, PubMed, and Cochrane Library databases in accordance with the search strategy. Title and abstract screening were used to exclude case reports, reviews, conference abstracts, editorials, letters, animal studies, and studies with irrelevant topics. Subsequently, 268 eligible studies were subjected to a full‐text review for further screening. Finally, 11 publications between 2018 and 2022 were included in the analysis.^20^, ^24^, ^25^, ^26^, ^27^, ^28^, ^29^, ^30^, ^31^, ^32^, ^33^ The flowchart depicting the process of search and identification of studies is shown in Figure 1.

### 3.2 Study characteristics and quality assessment

The study characteristics are summarized in Table 1. This meta‐analysis included 12 cohorts from 11 studies, totaling 932 patient samples ranging from 20 to 202 patients. Of these, four studies were conducted in Western countries, seven in Asian countries, and one across multiple regions. Regarding the type of ICI treatment, anti‐PD‐1 monotherapy was used in seven cohorts, whereas anti‐PD‐1/L1 or anti‐CTLA‐4 was used as monotherapy or in combination in five cohorts. Patients with esophageal cancer (EC), GC, and esophagogastric cancer were included in the study. WES and NGS were used for the analysis of blood and tumor tissue specimens. Regarding the sample source among the cohorts, eight used tumor tissue samples exclusively, two employed both tumor tissue and matched peripheral blood samples, and two relied solely on blood samples. Different studies have defined varying cutoff values for TML. PFS was used to express the survival data for 10 cohorts, whereas OS was used for eight cohorts. Based on the NOS score, five studies scoring 8–9 were considered high quality, and seven cohorts scoring 6–7 were considered moderate quality (Supplementary File S3).

**TABLE 1:** Characters of included studies in the meta‐analysis.

Reference	Region	Number of patients (high/low TMB)	Type of ICIs	Tumor type	Sample source	Sequencing method	TMB cutoff value	Median TMB value (range)	Survival outcome
Bai et al.^24^	Asian (China)	20 (13/7)	ICI	GC	Tumor	NGS	8.82 mut/Mb	NA	PFS
Greally et al.^25^	Western (USA)	89 (22/67)	ICI	EGC	Tumor	NGS	8.78 mut/Mb	5.6 mut/Mb	PFS, OS
Huang et al.^26^	Asian (China)	23 (11/12)	Anti‐PD‐1	EC	Tumor, blood	WES	60 mut/tumor	60 mut/tumor (15–219)	PFS
Kim et al.^27^	Asian (Korea)	105 (11/94)	Anti‐PD‐1	GC	Tumor	NGS	18.03 mut/Mb	NA	PFS, OS
Kim et al.^28^	Asian (Korea)	63 (8/55)	Anti‐PD‐1	GC	Tumor	NGS	14.31 mut/Mb	4.23 mut/Mb	PFS
Lu et al.^29^	Asian (China)	63 (11/52)	Anti‐PD‐1	GC (GEJC)	Tumor	NGS	8 mut/Mb	NA	PFS, OS
Mishima et al.^30^	Asian (Japan)	58 (34/24)	Anti‐PD‐1	GC	Tumor	NGS	10 mut/Mb	NA	PFS
Samstein et al.^31^	Western (USA)	126 (30/96)	ICI	EGC	Tumor	NGS	7.9 mut/Mb	7.4 mut/Mb	OS
Shitara et al.^20^	Multiple areas	202 (36/166)	Anti‐PD‐1	GC (GEJC)	Tumor	WES	175 mut/exome	NA	OS
Valero et al.^32^	Western (USA)	49 (12/37)	ICI	EC	Blood	NGS	8 mut/Mb	5.3 mut/Mb	PFS, OS
Valero et al.^34^	Western (USA)	71 (15/56)	ICI	GC	Blood	NGS	7 mut/Mb	4.4 mut/Mb	PFS, OS
Wang et al.^33^	Asian (China)	54 (12/42)	Anti‐PD‐1	GC (GEJC)	Tumor, blood	WES	12 mut/Mb	NA	PFS, OS

Abbreviations: EC, esophageal cancer; EGC, esophagogastric cancer; GC, gastric cancer; GEJC, gastroesophageal junction cancer; ICI, immune checkpoint inhibitor; mut, mutation; mut/Mb, mutation per megabase; NA, not available; NGS, next‐generation sequencing; OS, overall survival; PD‐1, programmed cell death protein 1; PFS, progression‐free survival; TMB, tumor mutation burden; WES, whole‐exome sequencing.

### 3.3 Key findings

**FIGURE 2:**
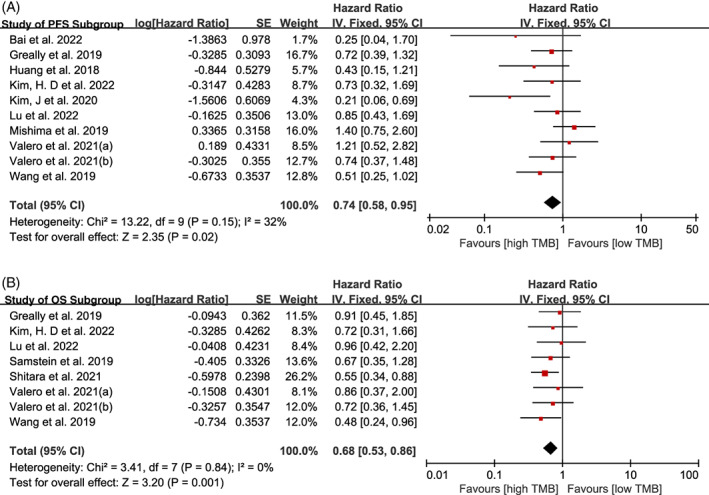
The forest plots of hazard ratios for progression‐free survival (PFS) (A) and overall survival (OS) (B) in patients with high tumor mutational load compared to those with low TML. CI, confidence interval; TMB, tumor mutational burden.

We performed a meta‐analysis of 10 cohorts for PFS and eight cohorts for OS, comparing groups of patients with high and low TML. A fixed‐effects model was used to calculate summary HRs with the corresponding 95% CIs. Our results showed that, as indicated by the summary HRs for PFS (HR = 0.74; 95% CI: 0.58, 0.95; *p* = .020) and OS (HR = 0.68; 95% CI: 0.53, 0.86; *p* = .001), the high TML group had a better survival benefit than the low TML group (Figure 2).

### 3.4 Publication bias

**FIGURE 3:**
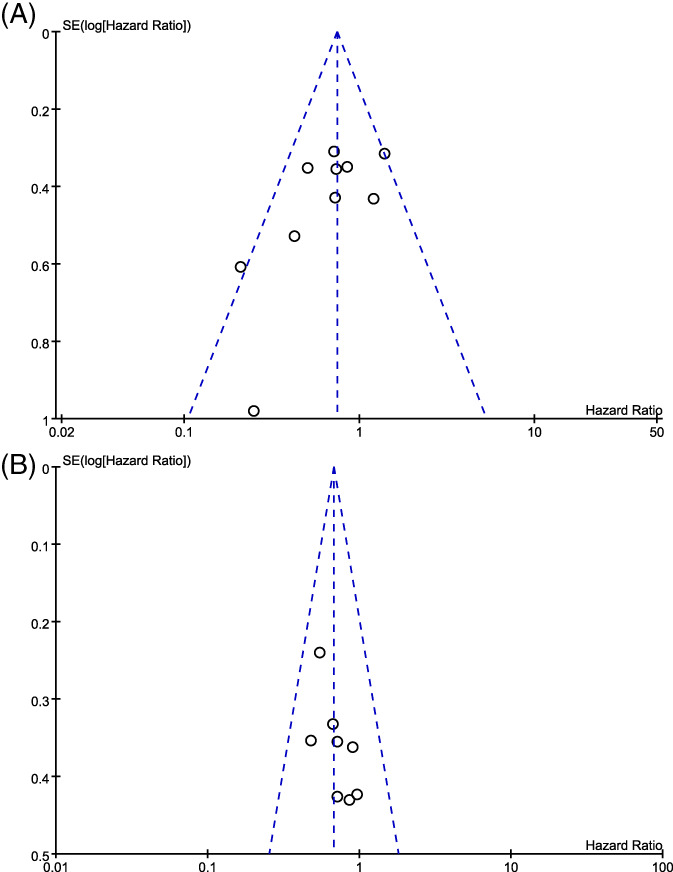
The funnel plots for pooled effects for progression‐free survival (A) and overall survival (B).

Funnel plots for publication bias analysis are shown in Figure 3. Publication bias was quantitatively assessed using Egger's test for PFS (*p* = .045). We reanalyzed the correlation after excluding one study with a sample size of 20, as publication bias appeared to be primarily driven by studies with small sample sizes. The results (pooled HR = 0.76; 95% CI: 0.59–0.97; *p* = .030) showed that PFS remained improved in the high TML group. Notably, publication bias disappeared (*p* Egger = .067). The absence of publication bias was demonstrated using Egger's test for OS (*p* = .062).

### 3.5 Assessment of heterogeneity and sensitivity analysis

**FIGURE 4:**
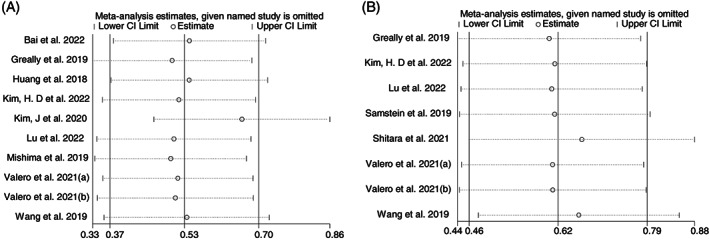
Sensitivity analysis of tumor mutational load with progression‐free survival (A) and overall survival (B). CI, confidence interval.

There was little heterogeneity in the summary HR for PFS among the combined studies (*I* ^2^ = 32%, *p* = .150) and no heterogeneity in OS (*I* ^2^ = 0, *p* = .840) between patients in groups with and without high TML. As shown in Figure 4, the sensitivity analysis showed that when the study reported by Kim et al.^28^ is excluded, the summary 95% CI of the remaining nine studies exceeded 1 (95% CI: 0.61, 1.01), suggesting no association of TML with PFS, contrary to our previous results. This suggests that this study significantly influenced the overall results and could be considered less robust. After exclusion, *I* ^2^ decreased to 4%. Sensitivity analysis of OS showed a minimal change in the pooled effect when each article was excluded individually. According to GRADE standards, the quality of evidence was low for both OS and PFS.

### 3.6 Subgroup analysis of PFS

Table 2 presents the results of PFS subgroup analyses. Regarding the subgroups based on patient recruitment area, the high TML group showed superior PFS (HR = 0.70; 95% CI: 0.51, 0.97; *p* = .030) in the Asian country subgroup, whereas there is no significant difference in the Western country subgroup (HR = 0.82; 95% CI: 0.55, 1.22; *p* = .320). The anti‐PD‐1 subgroup (HR = 0.66; 95% CI: 0.41, 1.06; *p* = .090) and the ICI subgroup (HR = 0.78; 95% CI: 0.52, 1.15; *p* = .480) were not statistically different. The correlation between TML level and PFS benefit was independent of the cancer type. PFS did not differ significantly between the groups with and without high TMLs, regardless of whether the specimens were taken from tumors or blood. With regard to the TML sequencing approach, we found that in the WES subgroup, PFS was superior in patients with high TML than in those with low TML, whereas no difference was observed in the NGS subgroup. In subgroup analysis based on TML cutoff, no significant PFS advantage was observed between patients with high TML and those with low TML, regardless of whether their TML cutoff was ≥10 mutations per megabase (mut/Mb). Meanwhile, subgroup analyses significantly reduced the heterogeneity among the subgroups of Western countries, ICI treatment, blood source, WES method, and a TML cutoff value of <10 mut/Mb. No significant heterogeneity was observed among the subgroups.

**TABLE 2:** Subgroup analysis of progression‐free survival between TMB high and TMB low groups.

Subgroup	Number of study	HR (95% CI)	*p*‐Value	*I* ^2^	Heterogeneity between subgroups
Region					
Asian	7	0.70 (0.51, 0.96)	.030	49%	*p* = .570
Western	3	0.82 (0.55, 1.22)	.320	0%	
Type of ICIs					
Anti‐PD‐1	6	0.66 (0.41, 1.06)	.090	53%	*p* = .600
ICI	4	0.78 (0.52, 1.15)	.480	0%	
Tumor type					
EC	2	0.75 (0.27, 2.07)	.580	56%	*p* = .860
GC	7	0.68 (0.45, 1.04)	.080	45%	
Sample source					
Tumor	6	0.79 (0.58, 1.09)	.150	48%	*p* = .690
Blood	2	0.90 (0.53, 1.54)	.700	0%	
Sequencing method					
WES	2	0.48 (0.27, 0.86)	.010	0%	*p* = .110
NGS	8	0.82 (0.62, 1.08)	.150	34%	
TMB cutoff value					
≥10 mut/Mb	4	0.64 (0.31, 1.30)	.210	68%	*p* = .580
<10 mut/Mb	5	0.79 (0.56, 1.12)	.190	0%	

Abbreviations: CI, confidence interval; EC, esophageal cancer; GC, gastric cancer; HR, hazard ratio; mut/Mb, mutation per megabase; NGS, next‐generation sequencing; PD‐1, programmed cell death protein 1; TMB, tumor mutational burden; WES, whole‐exome sequencing.

### 3.7 Subgroup analysis of OS

Table 3 shows the results of the OS subgroup analysis. Subgroup analysis by treatment type showed that anti‐PD‐1 therapy had better OS outcomes in patients in the high TML group (HR = 0.61; 95% CI: 0.44, 0.84; *p* = .002), whereas there was no significant difference in the ICI subgroup. In the GC subgroup, the OS benefit was greater in patients with high TML (HR = 0.62; 95% CI: 0.47, 0.84; *p* = .002). A higher TML correlated with longer OS (HR = 0.69; 95% CI: 0.51, 0.92; *p* = .010) when the specimen was tumor‐derived. The difference between the groups with higher and lower TML was statistically significant in the WES subgroup, but not in the NGS subgroup. The OS was longer in the high TML group when the TML cutoff value was ≥10 mut/Mb (HR = 0.57; 95% CI: 0.33, 0.97; *p* = .040). The association between TML levels and OS was independent of the region from which the patients were recruited. In the subgroups of EC, blood samples, and TML cutoff value <10 mut/Mb, the high TML group did not demonstrate any significant advantages over the low TML group. As shown in Table 3, no heterogeneity was observed within each subgroup or between corresponding subgroups.

**TABLE 3:** Subgroup analysis of overall survival between TMB high and TMB low groups.

Subgroup	Number of study	HR (95% CI)	*p*‐Value	*I* ^2^	Heterogeneity between subgroups
Region					
Asian	3	0.66 (0.42, 1.04)	.070	0%	*p* = .600
Western	4	0.77 (0.54, 1.10)	.160	0%	
Type of ICIs					
Anti‐PD‐1	4	0.61 (0.44, 0.84)	.002	0%	*p* = .330
ICI	4	0.77 (0.54, 1.10)	.160	0%	
Tumor type					
EC	1	0.86 (0.37, 2.00)	.730	0%	*p* = .480
GC	5	0.62 (0.47, 0.84)	.002	0%	
Sample source					
Tumor	5	0.69 (0.51, 0.92)	.010	0%	*p* = .710
Blood	2	0.77 (0.45, 1.33)	.350	0%	
Sequencing method					
WES	2	0.53 (0.36, 0.78)	.001	0%	*p* = .110
NGS	6	0.79 (0.58, 1.07)	.130	0%	
TMB cutoff value					
≥10 mut/Mb	2	0.57 (0.33, 0.97)	.040	0%	*p* = .280
<10 mut/Mb	5	0.80 (0.57, 1.11)	.180	0%	

Abbreviations: CI, confidence interval; EC, esophageal cancer; GC, gastric cancer; HR, hazard ratio; ICI, immune checkpoint inhibitor; mut/Mb, mutation per megabase; NGS, next‐generation sequencing; PD‐1, programmed cell death protein 1; TMB, tumor mutational burden; WES, whole‐exome sequencing.

## 4 DISCUSSION

Our findings suggest that, among patients with UGI cancers treated with ICIs, the high mutation load group exhibited better PFS and OS than the low mutation load group. Subgroup analysis further reveals that this survival advantage is particularly evident among Asian patients, those with GC, those treated with anti‐PD‐1, those whose samples were obtained from tumor tissue, and when a TML cutoff of ≥10 mut/Mb was used. As higher TML did not correlate with improved prognosis in non‐ICI‐treated patients, the improved OS and PFS in patients with high TML should be attributed to the efficacy of immunotherapy.^34^ Numerous other studies have also shown that elevated TML is linked to a favorable prognosis in patients with solid tumors undergoing immunotherapy.^14^, ^35^, ^36^

As previously mentioned, superior PFS was observed in Asian and GC subgroups. Recent large solid tumor data from an Asian population revealed that the TML of GC in the Advanced Origin Med cohort was significantly higher than that in the Memorial Sloan Kettering Cancer Center cohort, which may account for the clinical benefits observed in our study.^37^ This is because our study included a larger cohort of patients with GC and these patients were predominantly Asian. Our results demonstrated a significant OS benefit in patients with high TML who received anti‐PD‐1 therapy. In addition, promising results have been obtained with combination therapy with anti‐PD‐1/PD‐L1 and anti‐CTLA‐4.^38^ Furthermore, new immunotherapies—such as therapeutic vaccines and chimeric antigen receptor t‐cell therapy—are currently under investigation.^39^, ^40^ TML can serve as an indicator for screening individuals who may benefit from immunotherapies. Several trials, including CheckMate‐649, ATTRACTION‐04, and KEYNOTE‐062, have used immunotherapy in combination with chemotherapy or targeted therapies.^41^, ^42^, ^43^ In fact, nivolumab plus chemotherapy for the first‐line treatment of advanced GC has been approved by the Food and Drug Administration of the US.

Although WES is relatively accurate in measuring TML, NGS has gradually replaced it with the development of technology owing to its better economy and timeliness.^44^ Our results demonstrated that patients with high TML levels receiving ICI therapy detected by WES exhibited a survival advantage over those detected by NGS, which differs from a previous study.^36^ This may be due to the limited number of studies that used the WES method, resulting in less stable results. Compared to tumor tissue samples, blood samples showed no benefit in the ICI‐treated high TML group. This may be due to insufficient circulating tumor DNA in peripheral blood. However, it can be challenging to obtain sufficient tumor tissue for molecular testing in patients with advanced disease; therefore, a blood‐based method for the detection of TML is a viable option.^45^ The correlation between TML and blood‐based TML, as well as the synergistic effects of these predictive biomarkers on clinical outcomes, requires further investigation.^46^ While there may not be a universal definition of what constitutes high TML, a multi‐tumor clinical trial has shown that a high TML is associated with improved survival in patients receiving ICIs across a broad range of cancer types.^31^ Anti‐PD‐1 therapy was approved by the Food and Drug Administration of the US in 2020 for all solid tumor types with TML ≥10 mut/Mb based on Keynote 158 trial.^47^ The results of the cohort study further support the notion that, across cancer types, tumors with a TML ≥10 mut/Mb have a higher response rate to ICI therapy.^48^ For this reason, a cutoff of 10 mut/Mb was chosen. Our findings demonstrate that the summary HRs for TML cutoff values ≥10 mut/Mb are statistically significant. However, several challenges remain in integrating TML into standard clinical practice, including inconsistencies in its definition and the need for the standardization of analytical methods across laboratories and platforms.^49^

Although the prognostic impact of TML has been investigated in melanoma, lung, colorectal, and breast cancers, its association with survival outcomes in esophageal and GCs remains unknown.^17^, ^50^, ^51^, ^52^ Our meta‐analysis explored, for the first time, the relationship between TML and UGI tumors, providing a valuable research reference for the future use of TML as a potential predictive biomarker to guide clinical immunotherapy. However, our study has several limitations which require attention. Only English studies were considered in this review, which may have resulted in the omission of important literature published in other languages. The limited number of studies and small sample size may have impacted the accuracy of our findings. In addition, the correlation between tumor and blood TML has not been conclusively established, and the standardization of TML sequencing remains an issue. Therefore, our conclusions may be less stable and require further investigation. In addition to TML, other biomarkers used to predict immunotherapy efficacy include PD‐L1, MSI, and Epstein‐Barr virus. As the number of approved ICIs increases and the corresponding landmark trials for PD‐L1 antibodies use different immunohistochemical assays, the need for clinical and logistic harmonization remains unmet.^53^ MSI status and Epstein‐Barr virus positivity may serve as more effective predictive biomarkers for immunotherapy responses; however, the incidence rates of these biomarkers are relatively low.^54^ To date, no single biomarker has been able to accurately and universally predict the efficacy of immunotherapy. We believe that identifying the optimal combination of biomarkers for predicting immunotherapeutic responses represents a future direction. The most promising predictive combination of TML and other relevant biomarkers must be validated in large‐scale randomized trials.

## 5 CONCLUSION

Our meta‐analysis demonstrated that TML is a promising predictive biomarker, as high TML is significantly associated with longer survival in patients with UGI cancer who received ICIs. We suggest that 10 mut/Mb should be used as a cutoff value for the detection of TML to identify potential responders to immunotherapy.

## AUTHOR CONTRIBUTIONS

**Chenghao Ma:** Conceptualization (lead); data curation (lead); formal analysis (lead); investigation (lead); methodology (lead); project administration (lead); validation (lead); visualization (lead); writing – original draft (lead); writing – review and editing (lead). **Qiong Teng:** Data curation (equal); formal analysis (equal); investigation (equal); writing – original draft (equal). **Liang Shang:** Funding acquisition (equal); project administration (equal); resources (equal); supervision (lead); writing – original draft (equal). **Fengying Du:** Formal analysis (equal); software (equal); visualization (equal); writing – original draft (equal); writing – review and editing (equal). **Leping Li:** Funding acquisition (lead); project administration (equal); resources (lead); supervision (equal); writing – original draft (equal).

## CONFLICT OF INTEREST STATEMENT

All authors have completed the ICMJE uniform disclosure form. The authors have stated explicitly that there are no conflicts of interest in connection with this article.

## ETHICS STATEMENT

There were no ethical issues involved in our study.

## Supporting information


Supplementary File 1.

Supplementary File 2.

Supplementary File 3.

## Data Availability

Data Availability StatementThe original contributions presented in the study are included in the article. Further inquiries can be directed to the corresponding author.
