# Supplementary material for: Tumor mutation load better predicts the prognosis of patients treated with immune checkpoint inhibitors in upper gastrointestinal cancers: A systematic review and meta‐analysis
Source: Cancer Rep (Hoboken). 2024 Jan 11;7(2):e1959. doi: 10.1002/cnr2.1959 (PMC10849990; doi:10.1002/cnr2.1959)

**Pubmed (346 articles were retrieved.)**

(“Esophageal Neoplasms”[Mesh] OR **"Stomach Neoplasms"[Mesh] OR ("Upper Gastrointestinal Tract"[Mesh] OR ((upper[Tiab] AND (gastrointestinal[Tiab] OR GI[Tiab]) AND tract[Tiab]) OR Esophagus[Tiab] OR Esophageal[Tiab] OR Esophagogastric[Tiab] OR Gastroesophageal[Tiab] OR Stomach*[Tiab] OR Gastric[Tiab]) AND (Neoplasms[Mesh] OR Neoplas*[Tiab] OR Cancer*[Tiab] OR carcinoma*[Tiab] OR tumor*[Tiab] OR malignanc*[Tiab]))) AND ((****(mutation[Tiab] OR mutational[Tiab]) AND (burden[Tiab] OR load[Tiab])) OR TMB[Tiab] OR TML[Tiab]) AND** **(****"immunotherapy"[Mesh] OR immunotherap*[Tiab] OR immune checkpoint inhibitors[Mesh] OR ICI[Tiab] OR ICIs[Tiab] OR ICB[Tiab] OR ICBs[Tiab] OR ((PD-1[Tiab] OR Programmed Cell Death Protein 1[Tiab] OR PD-L1[Tiab] OR Programmed Death-Ligand 1[Tiab] OR CTLA-4[Tiab] OR Cytotoxic T-Lymphocyte-Associated Protein 4[Tiab] OR Checkpoint[Tiab]) AND (Inhibitor*[Tiab] OR Blocker*[Tiab] OR Blockade[Tiab] OR Inhibition[Tiab])) OR** **anti-PD-1[Tiab] OR "****Nivolumab"[Mesh] OR Nivolumab[Tiab] OR Opdivo[Tiab] OR** **pembrolizumab[Tiab] OR lambrolizumab[Tiab] OR Keytruda[Tiab] OR tislelizumab[Tiab] OR sintilimab[Tiab]** OR tyvyt**[Tiab] OR camrelizumab****[Tiab] OR carrelizumab[Tiab] OR cemiplimab[Tiab] OR libtayo[Tiab]** OR **penpulimab[Tiab] OR dostarlimab[Tiab] OR** **jemperli[Tiab] OR toripalimab[Tiab] OR pidilizumab[Tiab] OR anti-PD-L1[Tiab] OR atezolizumab[Tiab] OR Tecentriq[Tiab] OR durvalumab[Tiab] OR Imfinzi[Tiab] OR** **avelumab[Tiab] OR bavencio[Tiab] OR** **anti-CTLA-4[Tiab] OR "Ipilimumab"[Mesh] OR Ipilimumab[Tiab] OR Yervoy[Tiab] OR** **tremelimumab[Tiab] OR ticilimumab[Tiab])**

**Embase (761 articles were retrieved.)**


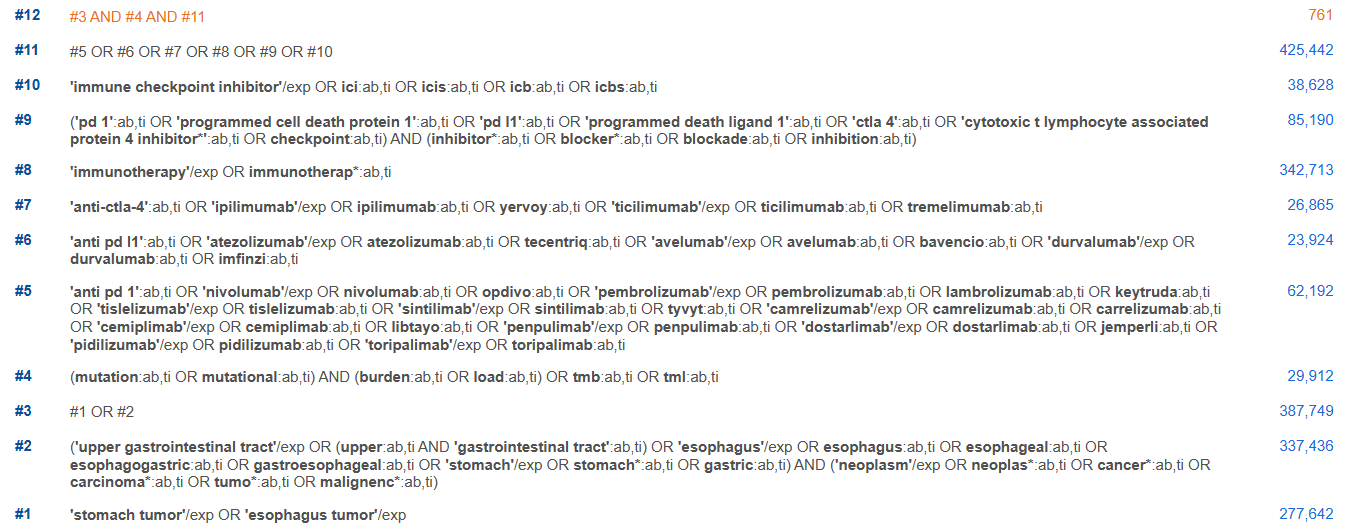


**Cochrane library (21 articles were retrieved.)**


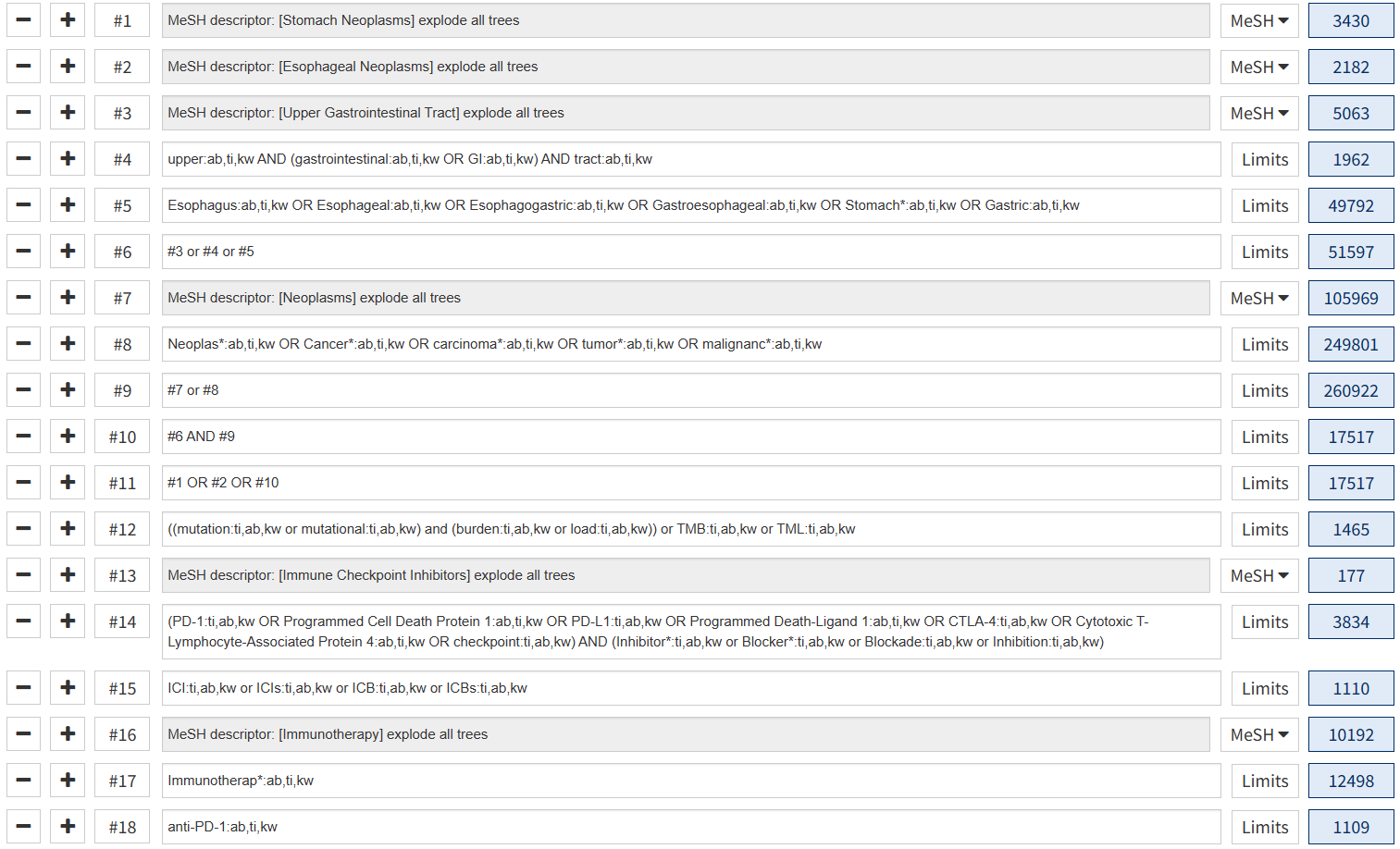

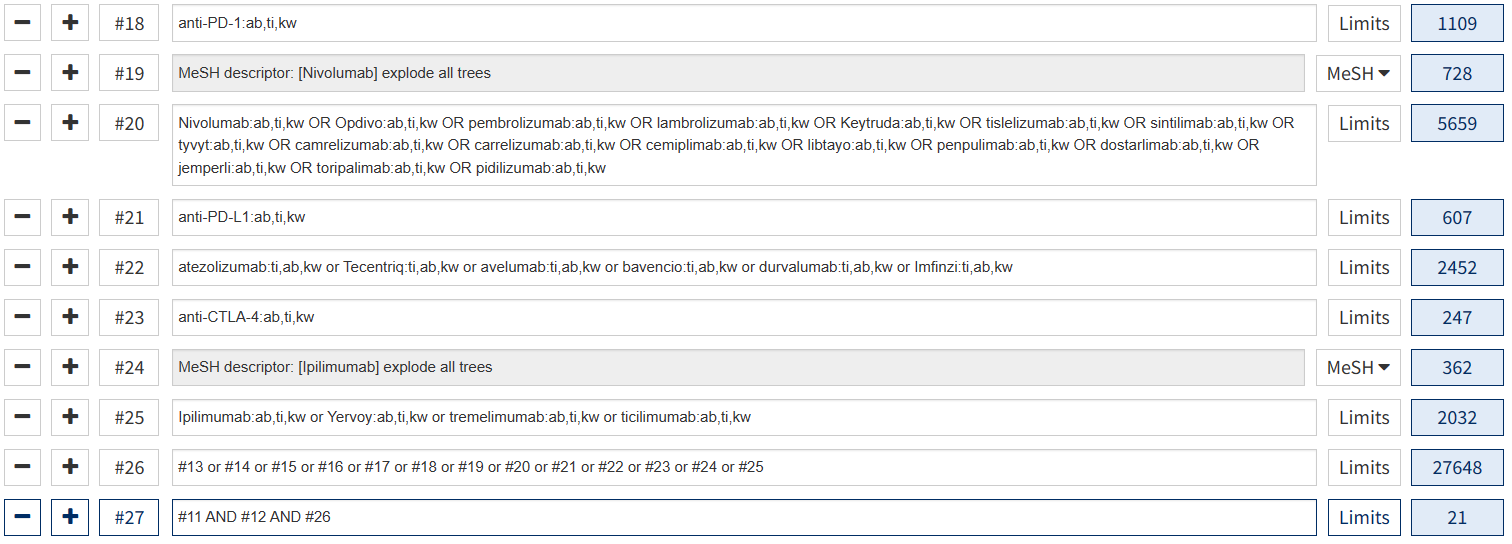


**Web of science (411 articles were retrieved.)**


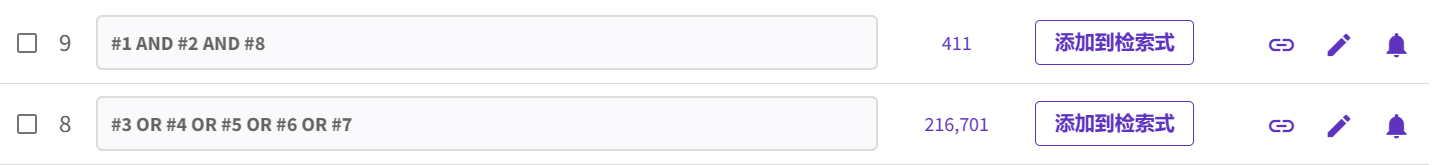


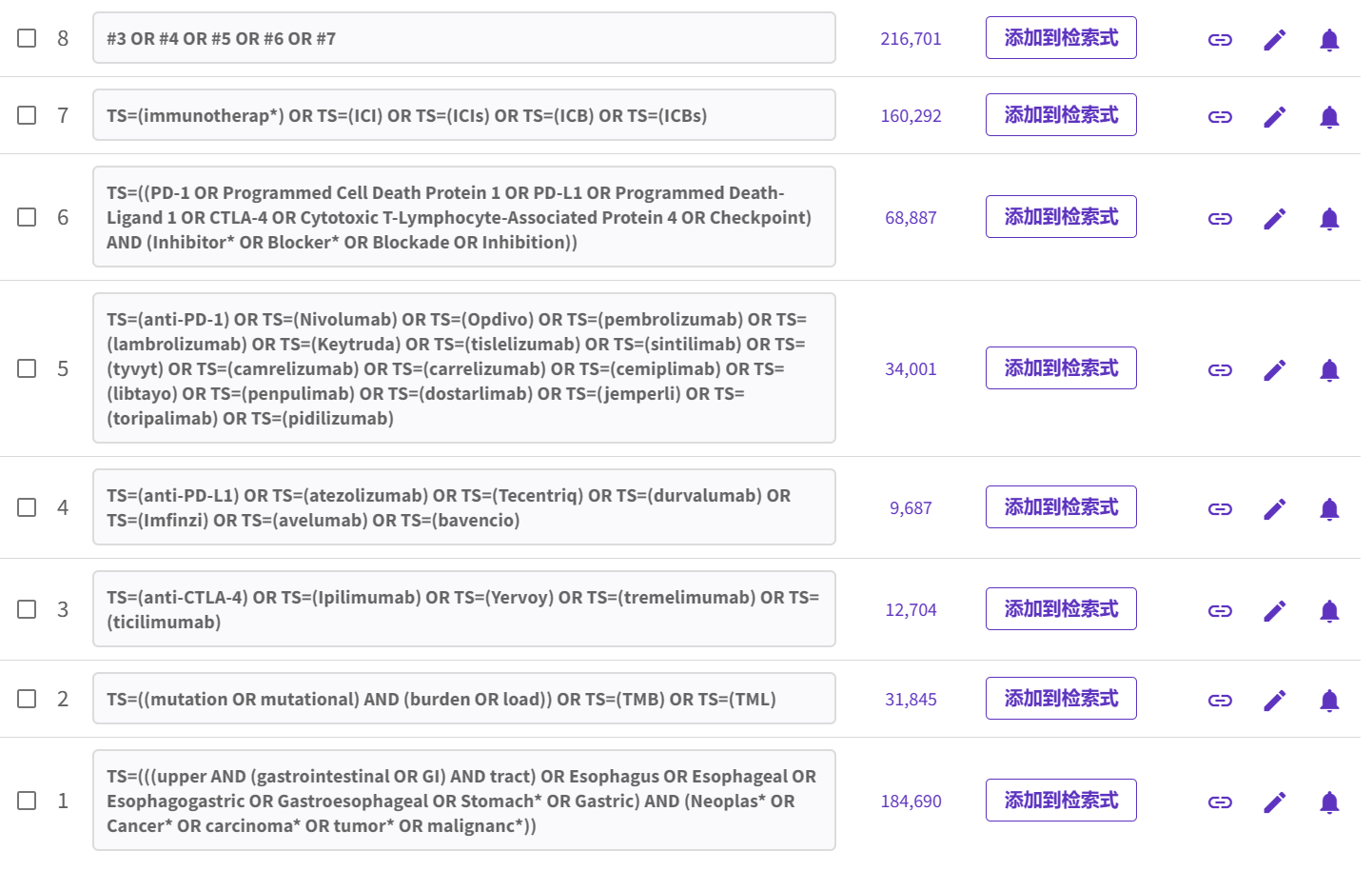

Supplement: Supplementary file 2 [file CNR2-7-e1959-s001.docx]
