# Supplementary material for: Tumor mutation load better predicts the prognosis of patients treated with immune checkpoint inhibitors in upper gastrointestinal cancers: A systematic review and meta‐analysis
Source: Cancer Rep (Hoboken). 2024 Jan 11;7(2):e1959. doi: 10.1002/cnr2.1959 (PMC10849990; doi:10.1002/cnr2.1959)
Supplement: Supplementary file 3 [file CNR2-7-e1959-s003.docx]

| Study | Selection | | | | Comparability | Outcome | | | NOS score |
| --- | --- | --- | --- | --- | --- | --- | --- | --- | --- |
|  | Q1 | Q2 | Q3 | Q4 | Q5 | Q6 | Q7 | Q8 |  |
| Bai et al. 2022 | / | * | * | * | / | * | * | * | 6 |
| Greally et al. 2019 | * | * | * | * | * | * | * | * | 8 |
| Huang et al. 2018 | * | * | * | * | / | * | * | * | 7 |
| Kim, H. D et al. 2022 | * | * | * | * | / | * | * | * | 7 |
| Kim, J et al. 2020 | / | * | * | * | ** | * | * | * | 8 |
| Lu et al. 2022 | * | * | * | * | / | * | * | * | 7 |
| Mishima et al. 2019 | * | * | * | * | * | * | * | * | 8 |
| Samstein et al. 2019 | * | * | * | * | ** | * | * | * | 9 |
| Shitara et al. 2021 | * | * | * | * | * | * | * | * | 8 |
| Valero et al. 2021(a) | * | * | * | * | / | * | * | * | 7 |
| Valero et al. 2021(b) | * | * | * | * | / | * | * | * | 7 |
| Wang et al. 2019 | / | * | * | * | / | * | * | * | 6 |

**TABLE S1.** Quality assessment of studies included in the meta-analysis using Newcastle-Ottawa Scale (NOS).

Q1: Representativeness of the exposed cohort; Q2: Selection of the non-exposed cohort; Q3: Ascertainment of exposure; Q4: Outcome of interest not present at start of study; Q5: Comparability of cohorts; Q6: Assessment of outcome; Q7: Follow-up long enough; Q8: Adequacy of follow up of cohorts.
